# Post-treatment haemolysis in severe imported malaria after intravenous artesunate: case report of three patients with hyperparasitaemia

**DOI:** 10.1186/1475-2875-11-169

**Published:** 2012-05-17

**Authors:** Thierry Rolling, Stefan Schmiedel, Dominic Wichmann, Dieter Wittkopf, Gerd-Dieter Burchard, Jakob P Cramer

**Affiliations:** 1 Department of Internal Medicine I, Section for Tropical Medicine and Infectious Diseases, University Medical Centre Hamburg-Eppendorf (UKE), Martinistrasse 52, 20246, Hamburg, Germany; 2 Department of Intensive Care, University Medical Centre Hamburg-Eppendorf (UKE), Martinistrasse 52, 20246, Hamburg, Germany; 3 Department of Transfusion Medicine, University Medical Centre Hamburg-Eppendorf (UKE), Martinistrasse 52, 20246, Hamburg, Germany; 4 Department of Clinical Research, Bernhard-Nocht-Institute for Tropical Medicine, Bernhard-Nocht-Strasse 74, 20359, Hamburg, Germany

**Keywords:** Severe imported malaria, Artesunate, Haemolysis, Reticulocytes

## Abstract

Parenteral artesunate has been shown to be a superior treatment option compared to parenteral quinine in adults and children with severe malaria. Little evidence, however, is available on long-term safety. Recently, cases of late-onset haemolysis after parenteral treatment with artesunate have been reported in European travellers with imported *Plasmodium falciparum* malaria. Therefore, an extended follow-up of adult patients treated for severe imported malaria was started in August 2011 at the University Medical Center Hamburg-Eppendorf. Until January 2012, three patients with hyperparasitaemia (range: 14-21%) were included for analysis. In all three patients, delayed haemolysis was detected in the second week after the first dose of intravenous artesunate. Reticulocyte production index remained inadequately low in the 7 – 14 days following the first dose of artesunate despite rapid parasite clearance. Post-treatment haemolysis after parenteral artesunate may be of clinical relevance in particular in imported severe malaria characterized by high parasite levels. Extended follow-up of at least 30 days including controls of haematological parameters after artesunate treatment seems to be indicated. Further investigations are needed to assess frequency and pathophysiological background of this complication.

## Background

Approximately 10,000 to 12,000 malaria cases imported to Europe are reported to the World Health Organization (WHO) annually [1]. In Germany, 617 cases of imported malaria were reported to the Robert Koch Institute in 2010, with an overall mortality rate of 0.3% (two cases) [2]. In a recent retrospective French study on patients with severe imported malaria – 97.8% of which were treated with intravenous quinine - mortality was determined to be 10.5% [3].

Parenteral artesunate has been shown to be a superior treatment option compared to parenteral quinine in adults and children with severe malaria in endemic countries [4,5]. In a large meta-analysis a significant reduction in mortality could be confirmed for adults (relative risk (RR) 0.61) and children (RR 0.76) treated with parenteral artesunate compared to parenteral quinine [6]. Artesunate showed a superior parasite clearance time compared to quinine. Artesunate is now the first-line treatment for severe malaria according to WHO guidelines [7].

Regarding short-term safety, hypoglycaemia occurred significantly less frequently in patients treated with artesunate. In children but not in adults, however, the incidence of neurological sequelae at the time of discharge was higher after treatment with artesunate compared to quinine which might be due to the fact that more severely diseased children survived. There was no difference discernible on long-term neurologic sequelae between artesunate and quinine arms 28 days after discharge in the African multicentre trial by Dondorp *et al.*[5,6]. Little evidence is available in particular on long-term safety due to trial designs and/or lacking infrastructure in endemic regions [8]. Very recently, cases of late-onset haemolysis have been reported retrospectively in European travellers with imported severe malaria [9]. The aetiology of this complication is unknown.

An extensive follow-up of patients treated for severe malaria to detect any uncommon findings and complications after parenteral artesunate was implemented at the University Medical Centre Hamburg-Eppendorf. From the beginning of this follow-up programme in August 2011 to January 2012, three patients with hyperparasitaemia were treated with parenteral artesunate, all of which developed late-onset haemolysis.

## Case presentation

### Patient 1

**Figure 1:**
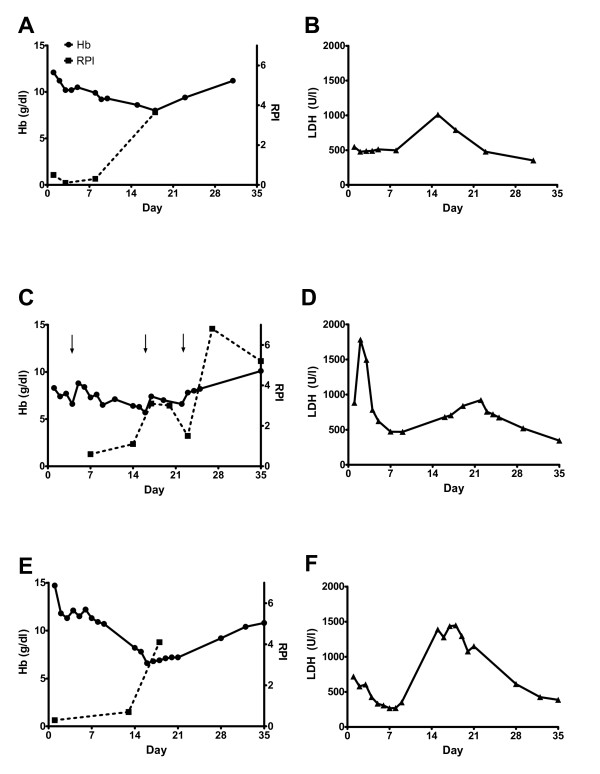
Time course of haemoglobin (Hb) levels / reticulocyte production index (RPI) as well as lactate dehydrogenase (LDH) levels in patient 1 (A and B), patient 2 (C and D, arrows indicating time points of transfusions) and patient 3 (E and F).

The first patient was a 19-year old German female without migrational background presenting without any co-morbidities or prior medication who stayed in Uganda for three weeks. She did not take any malaria prophylaxis. Four days after returning to Germany she developed fever and headaches. At the emergency department of a community hospital, she was diagnosed with uncomplicated malaria and showed 2% parasitaemia. She was transferred to a tertiary care centre specializing in tropical medicine for treatment of uncomplicated malaria. At the first evaluation in the emergency department (day 0), she did not fulfill any of the WHO criteria for severe malaria [7], and in line with the guidelines by the German Society of Tropical Medicine and International Health (DTG) treatment with mefloquine was initiated (starting with 750 mg) [10]. Over the next three to four hours, the patient’s clinical condition deteriorated. She became somnolent and her blood pressure dropped to 80/50 mmHg with a pulse rate of 108 bpm. At this time point, Giemsa-stained blood microscopy revealed a parasitaemia of 14%. The patient was now classified as complicated life-threatening malaria and was transferred to the intensive care unit. Intravenous artesunate (Guilin pharmaceutical factory, China) was given in four doses of 120 mg on day 0 and after 12, 24 and 48 hours equivalent to a total dose of 8mg/kg body weight. Because of thrombocytopenia of 15,000/μl (normal range: 150,000-400,000), she received one unit of packed thrombocytes. Indirect Coombs’ test was negative at this time. On day 1, her clinical condition improved strikingly and she was transferred to a regular ward on day 2. Anti-malarial treatment was continued orally with mefloquine (500 mg on day 2 followed by 250 mg eight hours later, equivalent to a total dose of 1500 mg including the initial dose of 750 mg on day 0). Parasitaemia declined rapidly from initially 14% to 10% after 12h and finally to less than 1% on day 3. On day 5, parasites were cleared. Two days later, the patient developed fever again and showed radiological signs of lung infiltration. Piperacillin-Sulbactam was started for hospital acquired pneumonia for 7 days. Hb dropped from 12 mg/dl (normal range: 12.3 - 15.3) at presentation to 10.2 mg/dl on day 2, stabilized between days 2 and 4 and decreased again in the following days to 8.6 g/dl on day 14. The decrease in Hb was accompanied by a second rise in lactate dehydrogenase (LDH) to 1010 U/l indicative of haemolytic anaemia (Figure 1A and 1B). Blood microscopy remained negative at this time point. Because of a slow but continuous clinical improvement she was discharged from hospital but follow-up was continued at the tropical medicine outpatient clinic. On day 16, the lowest Hb was measured but was accompanied with a significant rise in reticulocytes. Hb increased further during the following two weeks up to 11.2 g/dl (normal range: 12.3 - 15.3) on day 30 when the patient was without further clinical complaints. 

### Patient 2

The second patient was a 54-year old German male without migrational background returning from a one week-trip to Gambia. No malaria prophylaxis was taken. Prior medical history included surgically resected colorectal carcinoma two years earlier and arterial hypertension. His current medication consisted of metoprolol, hydrochlothiazide and felodipine for hypertension. Eight days after returning to Germany, he developed fever up to 41°C. The primary care physician diagnosed community acquired pneumonia and prescribed doxycycline for 7 days. After completing this course of antibiotic treatment, the symptoms still persisted and the patient became icteric. He presented himself to the emergency ward of a community hospital. Malaria was diagnosed and the patient was referred to a tertiary care hospital specializing in tropical medicine. Upon presentation at the tertiary care hospital (day 0), he appeared somnolent and showed hypotension of 100/50 mmHg with a pulse rate of 120 bpm. Hyperparasitaemia of 21% was diagnosed in Giemsa-stained microscopy. Furthermore, hyperbilirubinaemia (total bilirubin: 7.4 mg/dl, normal range: <1.2) and acute renal failure (creatinine: 3.4 mg/dl, normal range: 0.6 - 1.3; urea: 91 mg/dl, normal range: 8–26) were present. He was transferred to the intensive care unit and intravenous artesunate (Guilin Pharmaceutical Factory, China) was given in a total of four doses of 160 mg on day 0, after 12, 24 and 48 hours equivalent to a total dose of 9 mg/kg body weight. For haemodynamic support, he had to receive norepinephrine intermittently. On day 1, he received one unit of packed thrombocytes for thrombocytopenia of 7,000/μl (normal range: 150,000 - 400,000). Two units of packed red blood cells were transfused for anaemia at an Hb of 7.7 g/dl (normal range: 14.0 - 17.5) on day 3. Anti-malarial treatment was continued with oral atovaquone/proguanil on day 3 (three doses of 1000 mg atovaquone and 250 mg proguanil each) and on day 5 the patient was transferred to a regular ward. Parasite levels declined rapidly from 21% initially to 12% on day 1 and 1.5% on day 3. On day 7, blood film was negative. Two weeks after initial presentation at the tertiary care hospital, the Hb reached its lowest level of 5.7 g/dl (normal range: 14.0 - 17.5) with a concurrent rise in LDH, which peaked on day 21 (Figure 1C and D). Haptoglobin could not be detected consistent with haemolytic anaemia. No malaria parasites could be detected at this time point. The patient received an additional two units of packed red blood cells each on day 14 and on day 21. The patient was discharged on day 25. During follow-up, haemoglobin increased to 11.6 g/dl (normal range: 14.0 - 17.5) on day 49 while LDH decreased and the patient recovered clinically. This patient showed irregularities in the immune-haematological testing. On day 2, the indirect Coombs’ test was negative. On day 18, the indirect Coombs’ test was inconclusive while the direct Coombs’ test was positive for IgG specificity. On day 22, both indirect and direct Coombs test were negative again. On a repeated testing on day 23, indirect Coombs’ test was inconclusive and direct Coombs’ test was positive. The screening for complement factor C3d was negative. The specificity of the IgG antibodies could be shown to be anti-E with a very low titer of 1:1. The patient’s erythrocytes’ phenotype was A Rh pos CCD.ee Kell neg, none of the transfused units of packed red blood cells had Rh E phenotype.

### Patient 3

The third patient was a 55-year old German male (no migrational background) without any co-morbidities or prior medication. He returned from a business trip to Senegal and Gambia where he stayed for one week without malaria prophylaxis. Ten days after returning to Germany he developed multiple fever episodes up to 39°C and chills without any other complaints. He presented to a community hospital emergency department where the following laboratory findings were obtained: creatinine 3.6 mg/dl (normal range: 0.6 - 1.3), urea 43 mg/dl (normal range: 8–26) and total bilirubin 7.1 mg/dl (normal range: <1.2). Giemsa-stained microscopy revealed a parasitaemia of 20% and the patient was transferred to a tertiary care hospital specializing in tropical medicine for treatment of severe malaria. Surprisingly, the patient did not present any clinical complaints or symptoms upon first evaluation in the tertiary care hospital (day 0). Because of the laboratory diagnosis of severe malaria (hyperparasitaemia), a course of intravenous artesunate was provided in four doses of 240 mg on day 0, after 12, 24 and 48 hours, equivalent to a total dose of 9 mg/kg body weight which was switched to oral atovaquone/proguanil on day 3 (three doses of 1000 mg atovaquone and 250 mg proguanil each). Parasite levels declined rapidly from 20% initially to less than 1% on day 3. On day 9, the patient was discharged on his own explicit wish in good health condition. The patient’s initial Hb of 14.7 mg/dl decreased rapidly during the first day of hospitalization and was stabilized at 10.7 g/dl at discharge (normal range: 14.0 - 17.5) mg/dl; Figure 1 E). On the scheduled follow-up visit on day 13 in the tropical medicine clinic, a decrease in haemoglobin to 8.2 g/dl as well as a rise in LDH was detected and the patient was hospitalized again for further evaluation. Haemolysis was confirmed, haptoglobin was absent. The lowest haemoglobin was measured on day 15 with a value of 6.6 g/dl (normal range: 14.0 - 17.5), LDH peaked on day 17 (Figure 1E and 1F). Immune-haematological testing was performed on days 14 and 18. Direct and indirect Coombs’ tests were negative. No blood transfusions were given and the patient left the hospital against our strong advice on day 19. In the follow-up visits at his primary care physician, Hb levels were rising and LDH declined further while the patient’s clinical condition recovered fully (Figure 1E and 1F).

## Discussion

This case series describes three cases of late-onset haemolysis after parenteral artesunate for severe malaria in non-immune travellers returning from sub-Saharan Africa. In all three patients, an initial stabilization of Hb- and a decline of elevated LDH-levels in the first week after treatment initiation was seen. In the second week, Hb levels continued to decline while around day 14 a second rise in LDH levels was observed. At this time point haptoglobin was undetectable and total bilirubin rose again. Re-occurrence of biochemical markers compatible with delayed haemolytic anaemia 1–2 weeks after starting parenteral artesunate contrasted the rapid clinical improvement seen early after administering the first dose. Blood stage parasites were cleared around day 4–5 and all patients remained parasite-free during follow-up. One patient required blood transfusions. In all cases, haemolysis resolved slowly and Hb levels had not returned to normal levels 30 days after treatment initiation.

These findings are in line with a recent report by Zoller *et al.*[9]. Of 25 patients in seven centres for whom data were available, six developed a self-limiting episode of haemolytic anaemia, which was passively detected between days 15 and 32 (median: 15.5). Five of the patients required blood transfusions, all patients recovered fully.

As this case report is only descriptive and no control group (e.g. treated with quinine) exists, it is not possible to state with absolute certainty that the observed haemolysis is drug-related rather than disease-specific. The literature on longer-term follow-up of haematological parameters after severe malaria in general as well as in relation to specific antimalarial treatment is scarce. One report of 192 children treated with quinine showed a (clinically nonsignificant) drop in Hb after treatment and stabilization between days five and 21 [11]. The lack of a standardized follow-up precludes any definite statement on post-treatment haemolysis from this report. In decades of intensive use of quinine, no reports of post-treatment haemolysis have been published, which renders the existence of a comparable complication after quinine unlikely but not impossible.

Haemolytic anaemia is a characteristic finding in acute malaria. The aetiology of malarial anaemia is multifactorial and includes destruction of infected and uninfected erythrocytes as well as impaired erythropoiesis. Both, parasite toxicity as well as host immune mechanisms are causally involved [12]. Impaired erythropoiesis is reflected by a low reticulocyte production index (RPI) – which was also seen initially in the three patients described in this series (Figure 1). Usually, however, RPI rises concurrently with parasite clearance [13,14]. An adequate rise in RPI was delayed until the second week – long after parasites had been cleared and approximately at the time when haemolytic anaemia was diagnosed (Figure 1). Artemisinins have been reported to target erythropoiesis and to reduce reticulocyte counts several days after treatment [15,16] possibly explaining the late rise of RPI. Additionally, in the first patient the development of pneumonia could have prolonged inflammation-mediated impairment of erythropoiesis. No secondary infection can however explain the finding in the other two patients.

An explanation of a second peak of haemolysis might lie in the reduced life span of “pitted” erythrocytes. The number of these once-infected erythrocytes rises significantly after treatment with artesunate but not with quinine [17,18]. This mechanism would explain the correlation between delayed haemolysis and hyperparasitaemia, as the number of “pitted” erythrocytes is related to parasitaemia. This is supported by the facts, that i) all three patients had parasite levels of >10% infected red blood cells and that ii) all cases with post-treatment haemolysis described by Zoller *et al.* also had high parasite levels upon presentation (4 - 30%) [9].

Another potential explanation of late-onset haemolysis includes a delayed (re-)activation of pro-inflammatory reactions possibly triggered by the rapid and massive destruction of malaria parasite by artesunate and an increased presentation of parasitic antigens.

The fact that the patients described in this report received lower cumulative doses of artesunate than those in the publication by Zoller *et al.*[9] argues against a dose-dependent effect.

One (patient 2) of the two patients receiving immuno-haematological testing developed a positive Coombs’ test with IgG of anti-E specificity. This patient was the only one to receive packed red blood cell transfusions repeatedly after the first dose of artesunate. A delayed haemolytic transfusion reaction cannot be completely excluded in this patient, although it seems very unlikely. Delayed haemolytic transfusion reactions generally occur following alloimmunization after a previous transfusion. The time of onset is usually two to eleven days after the transfusion. The extent of haemolysis is mostly mild without clinical implications as only the transfused erythrocytes are being destroyed [19,20]. The titer of the detected antibody in our patient was extremely low and would not explain the amount of haemolysis seen in this patient. More important seems to be the fact that this patient showed the most severe malaria symptoms as well as the highest parasitaemia upon hospitalization (21%). In the case series by Zoller *et al.*, Coombs’ test was negative in all three patients in whom this test was performed [9]. All in all, this does not rule out immune-mediated haemolytic anaemia, but this has to be investigated in greater detail.

Two of the patients received drugs with anti-malarial activity prior to the first dose of artesunate. Patient 1 received 750 mg of mefloquine while patient 2 received a seven-day course of doxycycline for suspected bacterial infection. It is unclear whether these medications may have contributed to delayed haemolysis.

## Conclusions

After treating three hyperparasitaemic patients with parenteral artesunate, malaria parasites were cleared within a few days and the patients’ clinical condition improved rapidly. Post-treatment haemolysis, however, seems to be a relevant complication in non-immune travellers with imported malaria. Risk factors and pathophysiology are unknown. To gain statistically significant results for patients with imported severe malaria, data from cases at multiple centres will have to be accumulated in a standardized manner. Whether this complication also occurs in children with severe malaria in endemic regions is currently unknown. A regular follow-up of at least one month after treatment with parenteral artesunate including controls of haematological parameters seems to be indicated.

## Consent

Written informed consent was obtained from the patients for publication of this Case report. A copy of the written consent is available for review by the Editor-in-Chief of the journal.

## Competing interests

The authors declare having no competing interests.

## Authors' contributions

TR, JPC, DW and SS took part in the patients’ care. TR drafted the manuscript with contributions of JPC and GDB. All authors read and approved the final manuscript.
